# Association between spinal manipulative therapy and lumbar spine reoperation after discectomy: a retrospective cohort study

**DOI:** 10.1186/s12891-024-07166-x

**Published:** 2024-01-10

**Authors:** Robert J. Trager, Jordan A. Gliedt, Collin M. Labak, Clinton J. Daniels, Jeffery A. Dusek

**Affiliations:** 1 Connor Whole Health, University Hospitals Cleveland Medical Center, Cleveland, OH USA; 2 Department of Family Medicine and Community Health, School of Medicine, Case Western Reserve University, Cleveland, OH 44106 USA; 3 Department of Neurosurgery, Medical College of Wisconsin, Milwaukee, WI USA; 4 Department of Neurosurgery, University Hospitals Cleveland Medical Center, Cleveland, OH USA; 5 Rehabilitation Care Services, VA Puget Sound Health Care System, 9600 Veterans Drive, Tacoma, WA 98493 USA; 6 Department of Rehabilitation Medicine, University of Washington, Seattle, WA USA; 7 Susan Samueli Integrative Health Institute, University of California, Irvine, CA USA

**Keywords:** Chiropractic, Spinal manipulation, Lumbosacral region, Lumbar vertebrae, Surgical decompression, Intervertebral disc

## Abstract

**Background:**

Patients who undergo lumbar discectomy may experience ongoing lumbosacral radiculopathy (LSR) and seek spinal manipulative therapy (SMT) to manage these symptoms. We hypothesized that adults receiving SMT for LSR at least one year following lumbar discectomy would be less likely to undergo lumbar spine reoperation compared to matched controls not receiving SMT, over two years’ follow-up.

**Methods:**

We searched a United States network of health records (TriNetX, Inc.) for adults aged ≥ 18 years with LSR and lumbar discectomy ≥ 1 year previous, without lumbar fusion or instrumentation, from 2003 to 2023. We divided patients into two cohorts: (1) chiropractic SMT, and (2) usual care without chiropractic SMT. We used propensity matching to adjust for confounding variables associated with lumbar spine reoperation (e.g., age, body mass index, nicotine dependence), calculated risk ratios (RR), with 95% confidence intervals (CIs), and explored cumulative incidence of reoperation and the number of SMT follow-up visits.

**Results:**

Following propensity matching there were 378 patients per cohort (mean age 61 years). Lumbar spine reoperation was less frequent in the SMT cohort compared to the usual care cohort (SMT: 7%; usual care: 13%), yielding an RR (95% CIs) of 0.55 (0.35–0.85; *P* = 0.0062). In the SMT cohort, 72% of patients had ≥ 1 follow-up SMT visit (median = 6).

**Conclusions:**

This study found that adults experiencing LSR at least one year after lumbar discectomy who received SMT were less likely to undergo lumbar spine reoperation compared to matched controls not receiving SMT. While these findings hold promise for clinical implications, they should be corroborated by a prospective study including measures of pain, disability, and safety to confirm their relevance. We cannot exclude the possibility that our results stem from a generalized effect of engaging with a non-surgical clinician, a factor that may extend to related contexts such as physical therapy or acupuncture.

**Registration:**

Open Science Framework (https://osf.io/vgrwz).

**Supplementary Information:**

The online version contains supplementary material available at 10.1186/s12891-024-07166-x.

## Background

Lumbar discectomy is a surgical procedure often performed to remove herniated intervertebral disc material to alleviate refractory lower extremity pain, numbness, or weakness [1]. These symptoms, referred to in constellation as lumbosacral radiculopathy (LSR), may recur or persist following discectomy and prompt additional surgery (i.e., reoperation) [1–4]. Reoperation after lumbar discectomy is common, with a reported rate of 6 to 12% [1–4]. Reoperation within three months following discectomy is typically performed to address acute postoperative complications including hematoma or infection [2, 5]. However, the majority of reoperations are performed after three months to address recurrent same-level disc herniation [2, 6].

About 20% of patients undergoing lumbar discectomy have continued or recurrent LSR at one year after surgery [7]. For such cases, first-line treatments often consist of pain medications, spinal injections, cognitive behavioral therapy, and physical therapy exercises [8, 9]. In contrast, spinal reoperations are considered a final option due to generally having a lower success rate compared to primary surgeries [8]. In general, there is no consensus on the most appropriate care strategy for such patients [9].

Spinal manipulative therapy (SMT) has emerged as a potential treatment for those with ongoing LSR following spine surgery [10, 11]. Among patients receiving SMT from chiropractors, the primary providers of SMT in the United States (US) [12], approximately 11% of patients have a history of spine surgery [10]. Case series have reported improvements in pain and disability among patients with previous spine surgery receiving SMT [13–15]. However, to our knowledge, no studies have yet examined the association between SMT and spinal reoperation.

This study investigated the association between SMT and reoperation among patients experiencing LSR at least one year after lumbar discectomy. Considering the literature gap on effective treatments for LSR after discectomy and the limited but encouraging findings regarding SMT in this population [13–15], we hypothesized that adults receiving SMT for LSR at least one year after lumbar discectomy would be less likely to undergo reoperation compared to matched controls receiving usual care without SMT.

## Methods

### Study design

**Fig. 1:**
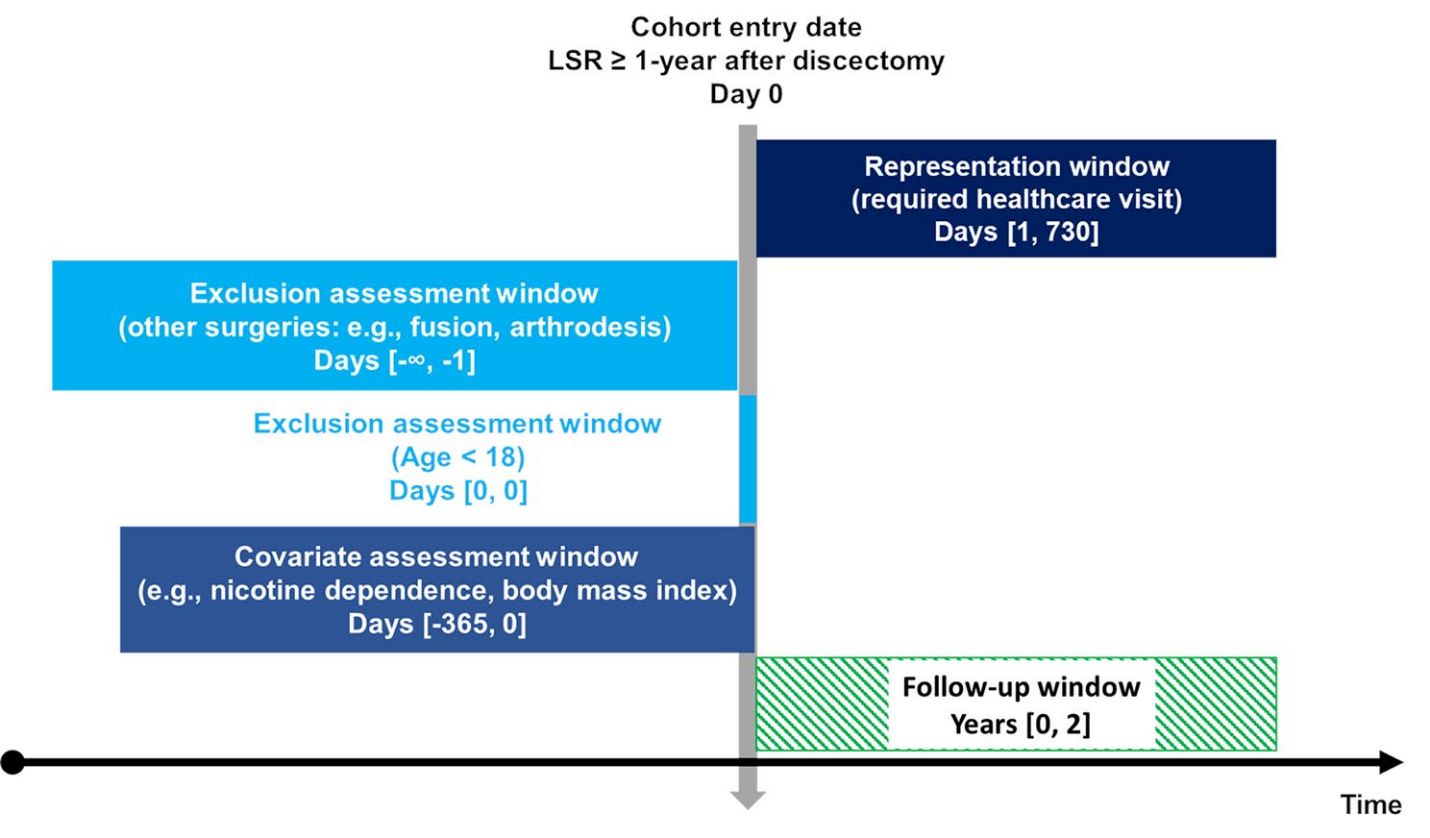
Study design. The vertical arrow represents the index date of enrollment. Windows to the left of this arrow represent preceding time windows over a span of days [#,#], while windows on the right indicate events following the index date of enrollment. The “∞” indicates that the time window reaches as far retrospectively as data are available. Abbreviations: lumbosacral radiculopathy (LSR). Figure adapted by Robert J. Trager using a Creative Commons template from Schneeweiss et al. [18]

This study used a retrospective cohort design based on a registered protocol [16]. We included patients from 20 years prior through two years prior to the query date of December 5, 2023, allowing for ascertainment of reoperations over a two-year follow-up (Fig. 1). Study reporting adheres to the Strengthening the Reporting of Observational Studies in Epidemiology (STROBE) guideline [17]. This study used de-identified data obtained from TriNetX (Cambridge, MA, USA) via its Clinical Research Center Honest Broker. Therefore, this study did not require approval from the University Hospitals Institutional Review Board (Cleveland, OH, USA) which considers the present study methods ‘not human subjects research’.

### Setting and data source

This study derived de-identified data from a US research network (TriNetX Inc., Cambridge, Massachusetts, USA), which aggregates health records data from over 114 million patients attending 80 academic medical centers and their affiliated hospitals and outpatient offices. The dataset includes both uninsured and insured patients. Data are regularly examined for completeness, plausibility, and conformance [19]. The TriNetX software automatically converts query codes to older versions when assessing older records [19] (e.g., International Classification of Diseases, 10th Edition [ICD-10] to 9th Edition codes). Although the specific participating healthcare institutions remain anonymous, chiropractors employed in integrative medical settings have over 20 years of clinical experience on average [20].

The natural language processing feature of TriNetX uses Averbis software (Averbis, Freiburg im Breisgau, DE), which employs machine learning and rules-based algorithms to extract meaning from unstructured clinical text while incorporating mechanisms to interpret context, intent, and negation [21, 22]. In previous studies, this software has demonstrated acceptable accuracy, reliability, and concordance with manual chart review in extracting clinical concepts related to diagnoses, symptoms, medications, and laboratory values [22, 23]. Specifically, studies estimated an overall Kappa value of 0.79 (good agreement) [22] and F1 values up to 0.89 representing the harmonic mean of recall and precision [22, 23]. However, performance of this software may vary across different clinical contexts. Our use of this technology aimed to facilitate our identification of patients with LSR, lumbar spine surgery (either prior for exclusion or for our outcome), and covariates for our propensity matching model.

### Participants

This study included adults at least age 18 years with ongoing LSR at least one year following lumbar discectomy. Eligibility criteria included patients with a diagnosis of lumbosacral radiculopathy and/or sciatica (Supplemental Table 1). This choice of diagnoses was intended to standardize cohorts to patients who would be potential candidates for reoperation [24], and reflected the most commonly used diagnoses for LSR [25]. To maximize data completeness and limit loss to follow-up, we required that patients have at least one additional health care encounter during the two-year follow-up window after the index date of inclusion.

Our definition of previous discectomy used codes from multiple standardized nomenclature systems (Supplemental Table 2) based on guidance from previous publications [26–28]. A broad definition of discectomy was used to maximize sample size including single or multiple level discectomy, via open, percutaneous, and endoscopic techniques. Patients who underwent discectomy as part of a disc arthroplasty (i.e., artificial disc replacement) or received an annular closure device were not included, as these represent more extensive procedures and may be managed differently with respect to SMT and/or reoperation.

We excluded patients with lumbar discectomy within one year preceding inclusion to eliminate patients with acute surgical complications (e.g., hematoma, infection ≤ 3 months postoperatively) who may require early revision surgery [2, 5]. In addition, eliminating patients who were in the first postoperative year was intended to foster between-cohort homogeneity. Considering that the incidence of lumbar spine reoperation remains relatively high during the first postoperative year [1, 2], inclusion of such patients could have led to relevant between-cohort differences. We also excluded patients with previous lumbar spine fusion, arthrodesis, spinal instrumentation, osteotomy, or surgery for spinal fracture or dislocation at any preceding time, as a strategy to help standardize the complexity of patients’ previous discectomy and clinical presentation. Patients in the usual care cohort were excluded from receiving chiropractic SMT on the index date of inclusion and throughout follow-up. Exclusionary criteria are summarized in Supplemental Table 3.

### Variables

We divided patients into two cohorts: (1) SMT; patients identified at the first instance of chiropractic SMT provided for LSR at least one year following lumbar discectomy, and (2) usual care; patients identified at an ambulatory visit for LSR at least one year following lumbar discectomy and not receiving chiropractic SMT. Patients with SMT were identified using Current Procedural Terminology codes indicating administration of SMT by chiropractors (98,940, 98,941, and 98,942) [29]. We chose to examine SMT performed by chiropractors, rather than other clinicians (e.g., osteopaths, physical therapists), because (1) it is specifically identifiable by procedure code [29], and (2) chiropractors are the most frequent providers of SMT in the US [30].

In an aim to minimize bias, we used propensity matching to control for variables present within one year anteceding inclusion and either positively or negatively associated with lumbar reoperation after discectomy. These included: age (negative [6, 31]), body mass index (positive; [32–34]), nicotine dependence (positive; [35]), and spondylolisthesis (positive; [36]) (Supplemental Table 4). Considering prior studies found no association between sex and race/ethnicity and reoperation [31, 32, 34, 36–39], we did not match for these variables but reported them for descriptive purposes. We added several markers of potential interventions for LSR following lumbar discectomy for descriptive purposes including chiropractic spinal manipulation, physical therapy evaluation, epidural steroid injection, transforaminal steroid injection, and medications including gabapentinoids, NSAIDs, opioids, and skeletal muscle relaxants [8, 10].

We examined a range of lumbar spine surgeries targeting degenerative disorders (e.g., disc herniation, stenosis) occurring within a two-year follow-up after the index date of inclusion. As guided by previous literature, these procedures included laminectomy, laminotomy, discectomy, fusion, and disc arthroplasty [26–28, 40] (Supplemental Table 5). We did not consider spinal cord stimulator procedures, radiofrequency ablation, annuloplasty, chemical ablation, percutaneous lysis of epidural adhesions, cementoplasty, corpectomy, and surgical procedures intended for spinal deformity (e.g., scoliosis, kyphosis), malignancy, infection, or hematoma within the definition of lumbar spine surgery for the purpose of this study. These procedures have different indications, and their inclusion could lead to relevant between-cohort bias.

### Statistical methods

We conducted statistical analysis using software available within the TriNetX platform. We compared baseline characteristics using an independent samples t-test for continuous variables or Pearson chi-squared test for categorical variables. Propensity scores to predict cohort assignment were calculated via logistic regression using Python (scikit-learn version 1.3 [Python Software Foundation, Delaware, USA]). This model estimated the log odds of receiving usual care as a linear combination of matched covariates. The fitted model generated a propensity score for each patient ranging from 0 (indicating the lowest likelihood of receiving usual care) to 1 (indicating the highest likelihood). To create balanced cohorts, patients were then matched using greedy nearest neighbor matching with a 1:1 ratio and a caliper width of 0.1 pooled standard deviations of the logit of the propensity score.

We assessed covariate balance using standardized mean difference, wherein absolute values of > 0.1 were considered to indicate residual imbalance [41–43], later adding *P*-values for additional context (*P* < 0.05 indicating potential imbalance). We reported the mean number of data points per patient per cohort and percentage of unknown demographic variables as markers of data density and completeness. A propensity score density graph was used to evaluate the success of matching. The risk ratio for reoperation was calculated both before and after matching by dividing cumulative incidence in the SMT cohort by the usual care cohort. We conducted a sensitivity analysis to examine the timing of reoperation by graphing cumulative incidence. For the SMT cohort, we also calculated the odds of a follow-up SMT visit and mean and median number of follow-up SMT visits. We used R (version 4.2.2, Vienna, AT [44]) and ggplot2 package [45] to plot cumulative incidence and propensity score density.

### Study size

Given the lack of prior similar studies to help generate a sample size estimate, we calculated a total required sample size of 712 using previous estimates of the incidence of reoperation (i.e., 6–12%) [1–4]. Using a z-test via GPower (Kiel University, DE), we powered the study to examine for a difference in incidence between cohorts of 6% (i.e., 6% vs. 12%), using an α-error of 0.05, power of 0.80, two tails, and allocation ratio of one.

## Results

### Participants

We identified eligible patients from multiple health care organizations (SMT: 4; usual care: 18). Before propensity matching there were 380 patients in the SMT cohort and 1,931 in the usual care cohort. After matching, each cohort had 378 patients (mean age 61 years). Regarding propensity-matched variables, before matching, patients in the SMT cohort were older and less often had nicotine dependence (SMD > 0.1, *P* < 0.05; Table 1). The proportion of patients with spondylolisthesis was low in both cohorts yet difficult to compare due to TriNetX’s feature of rounding up small counts to 10. Most propensity-matched variables were optimally matched (SMD < 0.1), while body mass index was adequately matched (SMD = 0.107; *P* > 0.05) [43]. Regarding descriptive (i.e., unmatched) variables after matching (Table 1): Prior use of lumbar magnetic resonance imaging, emergency department visits, and use of nonsteroidal anti-inflammatory drugs were similar between cohorts, (SMD < 0.1 and/or *P* > 0.05). Prior computed tomography, physical therapy evaluation, and lumbar epidural steroid injection appeared similar between cohorts, yet a direct comparison was hindered because of too few patients. Prior prescription of opioid analgesics, skeletal muscle relaxants, gabapentinoids, and receipt of transforaminal injection was greater in the usual care cohort whereas use of SMT was significantly greater in the SMT cohort (SMD > 0.1 and/or *P* < 0.05).

**Table 1:** Baseline characteristics before and after propensity matching

Variable	SMT	Usual medical care	SMD (*P*-value)	SMT	Usual medical care	SMD (*P*-value)
N	380	1,931		378	378	
Age*	60.8 (14.6)	56.5 (14.9)	0.292 (< 0.001)	60.8 (14.6)	60.9 (14.3)	0.009 (0.904)
BMI*	30.6 (6.3)	30.4 (6.5)	0.024 (0.73)	30.6 (6.3)	30.0 (6.3)	0.107 (0.226)
Female	239 (63%)	998 (52%)	0.228 (< 0.001)	237 (63%)	194 (51%)	0.231 (0.002)
Male	141 (37%)	932 (48%)	0.227 (< 0.001)	141 (37%)	183 (48%)	0.226 (0.002)
Not Hispanic or Latino	343 (90%)	1704 (88%)	0.065 (0.258)	341 (90%)	342 (90%)	0.009 (0.902)
Hispanic or Latino	≤ 10 (3%)	30 (2%)	0.075 (0.141)	≤ 10 (3%)	≤ 10 (3%)	0 (1)
American Indian or Alaska Native	0 (0%)	11 (1%)	0.107 (0.14)	0 (0%)	≤ 10 (3%)	0.233 (0.001)
Asian	0 (0%)	28 (1%)	0.172 (0.018)	0 (0%)	≤ 10 (3%)	0.233 (0.001)
Black or African American	0 (0%)	27 (1%)	0.168 (0.02)	0 (0%)	≤ 10 (3%)	0.233 (0.001)
Native Hawaiian or Other Pacific Islander	0 (0%)	13 (1%)	0.116 (0.109)	0 (0%)	≤ 10 (3%)	0.233 (0.001)
White	343 (90%)	1683 (87%)	0.098 (0.092)	341 (90%)	340 (90%)	0.009 (0.903)
Other race	≤ 10 (3%)	≤ 10 (1%)	0.170 (< 0.001)	≤ 10 (3%)	≤ 10 (3%)	0 (1)
Nicotine dependence*	33 (9%)	271 (14%)	0.169 (0.005)	33 (9%)	35 (9%)	0.018 (0.799)
Spondylolisthesis, lumbar*	≤ 10 (3%)	33 (2%)	0.063 (0.224)	≤ 10 (3%)	≤ 10 (3%)	0 (1)
Spondylolisthesis, lumbosacral*	≤ 10 (3%)	≤ 10 (1%)	0.170 (< 0.001)	≤ 10 (3%)	≤ 10 (3%)	0 (1)
Chiropractic manipulative treatment	310 (82%)	114 (6%)	2.359 (< 0.001)	309 (82%)	39 (10%)	2.054 (< 0.001)
Computed tomography, lumbar spine	≤ 10 (3%)	65 (3%)	0.043 (0.46)	≤ 10 (3%)	14 (4%)	0.060 (0.407)
Magnetic resonance imaging, lumbar	56 (15%)	251 (13%)	0.05 (0.361)	55 (15%)	57 (15%)	0.015 (0.838)
Physical therapy evaluation	23 (6%)	60 (3%)	0.141 (0.005)	21 (6%)	10 (3%)	0.147 (0.044)
Lumbar or sacral transforaminal steroid injection	19 (5%)	130 (7%)	0.074 (0.209)	18 (5%)	30 (8%)	0.130 (0.073)
Lumbar or sacral epidural steroid injection	≤ 10 (3%)	22 (1%)	0.110 (0.023)	≤ 10 (3%)	≤ 10 (3%)	0 (1)
Emergency visit	77 (20%)	400 (21%)	0.011 (0.842)	77 (20%)	71 (19%)	0.040 (0.582)
NSAIDs	193 (51%)	992 (51%)	0.012 (0.835)	192 (51%)	214 (57%)	0.117 (0.109)
Opioid analgesics	187 (49%)	1087 (56%)	0.142 (0.011)	187 (49%)	225 (60%)	0.203 (0.006)
Skeletal muscle relaxants	84 (22%)	553 (29%)	0.151 (0.009)	84 (22%)	108 (29%)	0.146 (0.045)
Gabapentinoids	65 (17%)	406 (21%)	0.100 (0.083)	65 (17%)	93 (25%)	0.183 (0.012)

Abbreviations: Body mass index (BMI); nonsteroidal anti-inflammatory drugs (NSAIDs); standardized mean deviation (SMD); spinal manipulative therapy (SMT). Chiropractic manipulative therapy includes chiropractic SMT as well as extraspinal manipulation provided by a chiropractor

* Propensity-matched variables (other variables reported for descriptive purposes)

### Descriptive data

**Fig. 2:**
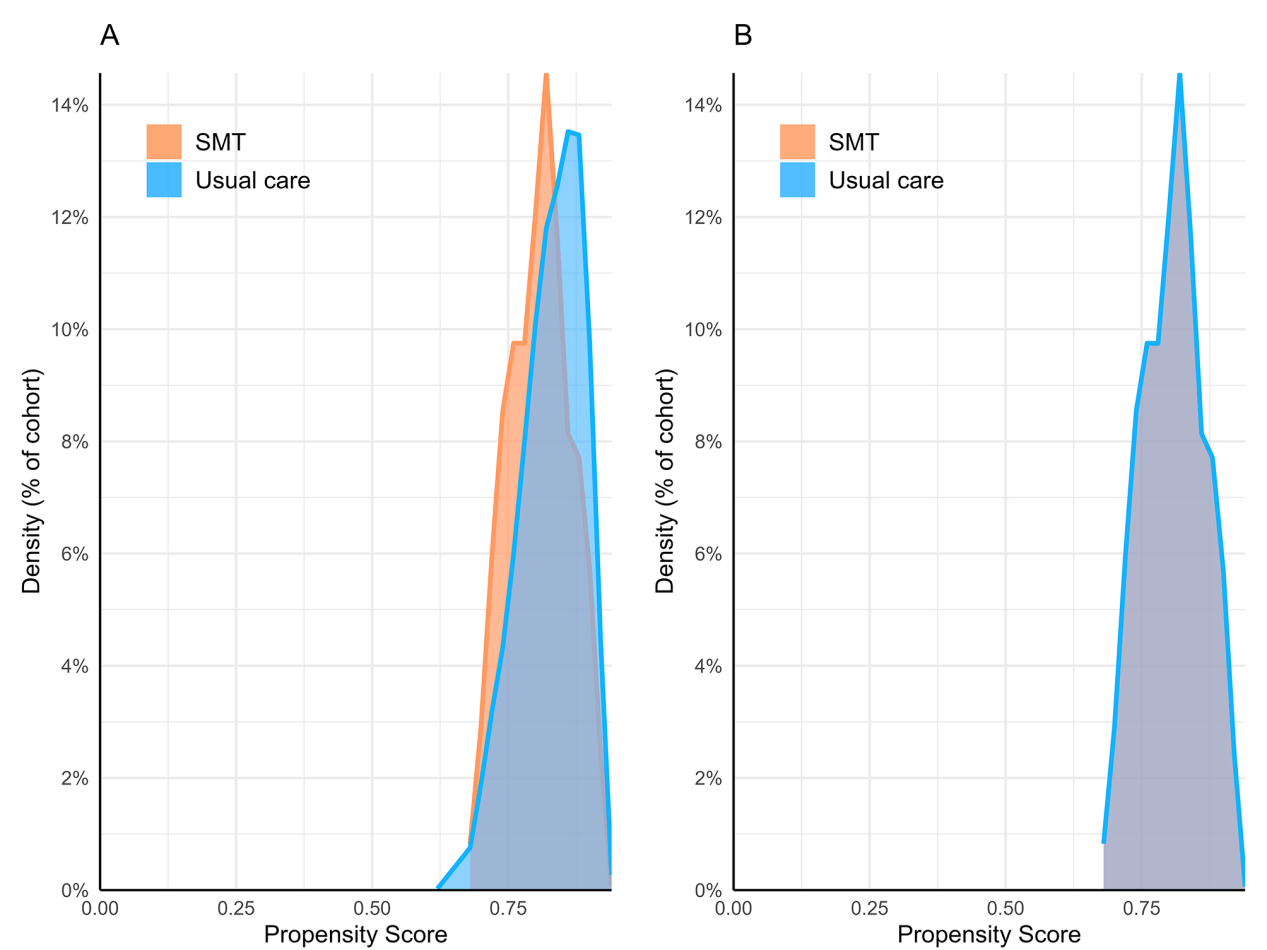
Propensity score density graph. Propensity scores before (**A**) and after (**B**) matching. The orange line and fill represent the spinal manipulative therapy (SMT) cohort while the blue line and fill represent the usual care cohort. Following matching, the propensity score densities overlap suggesting adequate balance of covariates

The mean number of data points per patient per cohort was sufficiently high (SMT: 9,442; usual care: 6,901). Following propensity matching, the occurrence of unknown demographic variables was comparable in both cohorts: unknown ethnicity (SMT: 9%; usual care: 9%, SMD = 0.009), unknown sex (0%, SMD = 0), and unknown age (0%, SMD = 0). After matching, the propensity score densities of each cohort were overlapping when graphed, indicating a sufficient balance of covariates (Fig. 2). The length of patients’ records before matching was at least 5 years for 100% of the CSM cohort and 99% of the usual care cohort when considering the span of available data before and after the index date. These results indicate minimal differences between the cohorts in terms of data density, data completeness, and covariate balance.

### Primary outcome

The incidence of lumbar spine reoperation over two years following the index date of inclusion was lower in the SMT cohort compared to the usual care cohort (Table 2). After propensity matching, 7% of the SMT cohort had undergone lumbar spine reoperation, compared to 13% of the usual care cohort, translating to an RR (95% CI) of 0.55 (0.35–0.85; *P* = 0.0062).

**Table 2:** Key results before and after propensity score matching

	Before matching		After matching	
	SMT	Usual care	SMT	Usual care
Number of patients	380	1,931	378	378
Lumbar spine reoperation N (%)	28 (7%)	268 (14%)	28 (7%)	51 (13%)
RR (95% CI)	0.53 (0.37–0.77; *P* < 0.0005)	(reference)	0.55 (0.35–0.85; *P* = 0.0062)*	(reference)

Abbreviations: spinal manipulative therapy (SMT), risk ratio (RR), 95% confidence intervals (95% CI)

* primary outcome

### Secondary outcomes

**Fig. 3:**
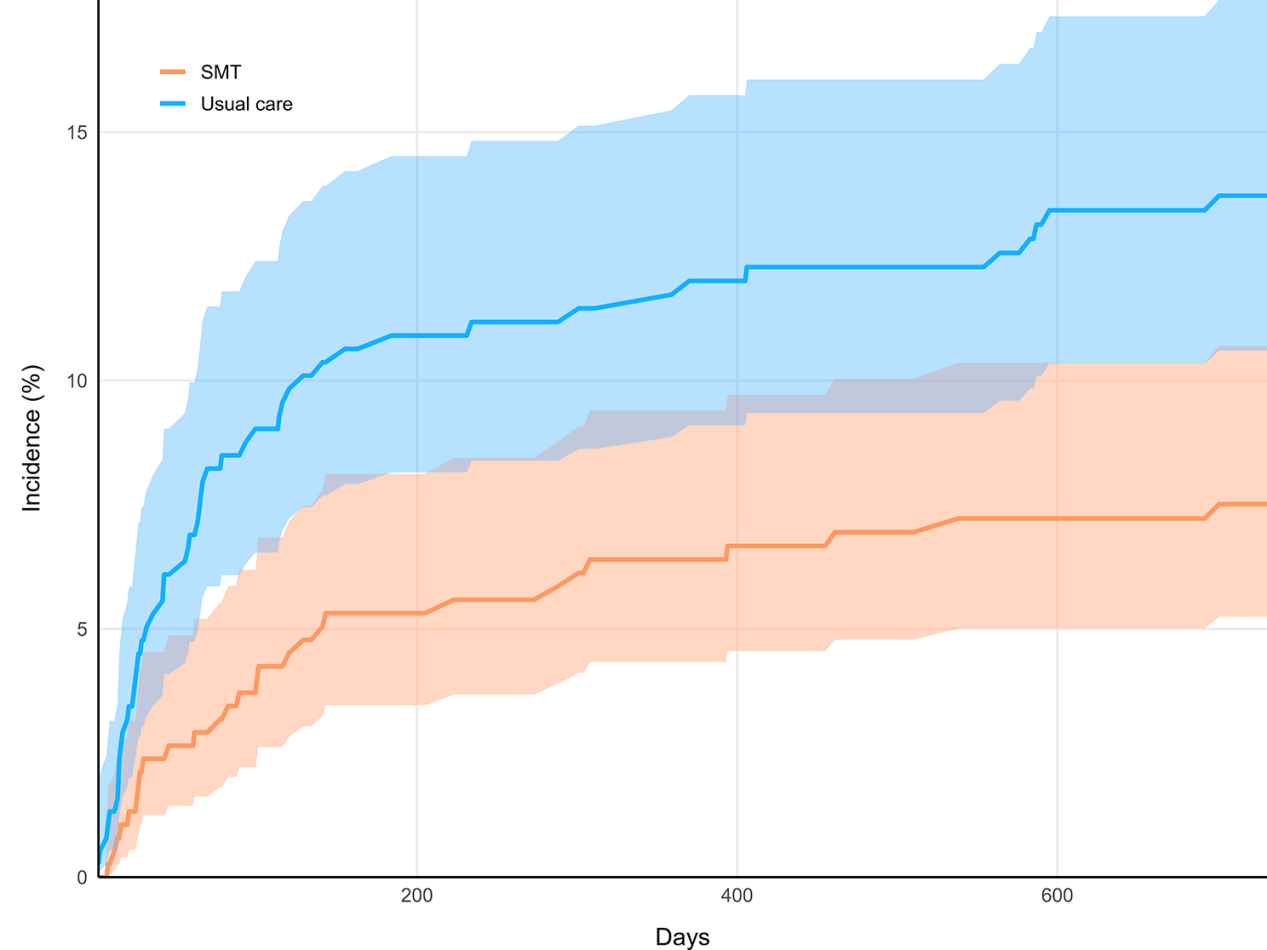
Cumulative incidence graph. Incidence curves for lumbar spine reoperation in the spinal manipulative therapy cohort (SMT; orange) and usual care cohort (blue) are shown over the two-year follow-up period (730 days). Shaded regions indicate 95% confidence intervals

In a cumulative incidence plot (Fig. 3), both cohorts’ incidence of reoperation increased in a curvilinear manner, with more reoperations occurring earlier during follow-up (< 200 days) then tapering. However, the usual care cohort incidence deviated early and remained elevated compared to the SMT cohort. The plotted cumulative incidences did not overlap or intersect, however there was overlap in the 95% confidence intervals. This overlap is suggestive of imprecision in the incidence estimates at each measured time point (i.e., each day of follow-up). The likelihood of reoperation over the entire follow-up window is best examined by the summary measure of the RR, which is our primary outcome.

After matching, most patients in the SMT had at least one follow-up SMT visit (*N* = 272; 72%). Including patients with zero follow-up SMT visits, the mean number of additional SMT visits was 11.9 (SD = 16.0), with a median of 6. Patients with at least one SMT follow-up had a median of 10 follow-up SMT visits. Given that 28% of patients had only a single SMT session and the mean number of follow up SMT sessions exceeded the median, along with a large SD, it suggests a positively skewed distribution of SMT visits. While direct normality testing is not possible given the constraints of the dataset, these findings suggest that a minority of patients receiving SMT had a much greater number of SMT visits than others.

## Discussion

To our knowledge, this is the first large-scale study to examine the association between SMT and spinal reoperation. The study findings support our hypothesis that adults with LSR receiving SMT at least one year after lumbar discectomy are less likely to undergo lumbar spine reoperation compared to matched controls not receiving SMT. A cumulative incidence graph demonstrated that surgeries tended to occur earlier in the two-year follow-up window in both cohorts.

In addition, it remains unclear whether mechanisms of SMT on pain or disability alone would account for our findings. It is possible that nonspecific therapeutic factors related to education and advice, the clinician-patient relationship, continuity of care, patient expectations, or avoidance of surgeon visits in the SMT cohort accounted for the observed reduction in lumbar spine reoperation [46, 47]. With these considerations, follow-up studies might include other comparator cohorts such as physical therapy exercises, acupuncture, or psychological therapies to broadly explore the potential impact of conservative care on reoperation. In addition, a qualitative study could explore patients’ reasons for pursuing or avoiding lumbar spine reoperation.

Our findings are consistent with prior research suggesting that patients receiving SMT for a new diagnosis of LSR are less likely to undergo discectomy [48]. However, our study population differed in that patients had undergone a discectomy previously. Nonetheless, the underlying rationale for this finding may be similar, as SMT has demonstrated efficacy in reducing pain and disability associated with LSR in two clinical trials [49, 50]. We therefore suspect that SMT could reduce the likelihood of reoperation after discectomy by alleviating symptoms of LSR.

While our study did not directly measure costs, the observed decrease in lumbar spine reoperations in the SMT cohort may translate to potential cost savings. Lumbar spine reoperations incur substantial costs (2017: $11,161 per patient; approximately $13,750 in 2023, per the US Bureau of Labor Statistics) [1]. In contrast, the costs of a single chiropractic visit are relatively lower (2023: $65 [51]) yet would be additive over the duration of care. Future investigations using administrative claims data could be used to comprehensively compare total costs, including those incurred from adjunctive therapies, modalities, and medications over the duration of care, which would be missed in our current simplified model. This would provide an improved understanding of whether SMT offers cost-effective benefits in a population at heightened risk of substantial reoperation costs.

SMT is generally considered safe, with severe adverse events occurring at an incidence ranging from less than one to seven in 100,000 treatments [52, 53]. However, there is a lack of studies examining the likelihood of SMT-related adverse events among patients with prior spine surgery [12, 54]. Although our study did not focus on identifying adverse events, our findings of reduced lumbar spine reoperation in adults receiving SMT serve as a potential marker of safety of this treatment, suggesting that SMT may be unlikely to contribute to recurrent disc herniation requiring reoperation. However, our findings do not inform clinicians if or when it is safe to administer SMT in patients with previous discectomy, due to included patients being uniformly at least one-year post-operation, lack of a direct measure for adverse events, and lack of detailed patient-level data. Further study is needed to examine potential adverse events more rigorously among patients with prior spine surgery receiving SMT.

While our findings are promising, it is important to emphasize the necessity for well-designed prospective studies to further investigate this topic. Our study offers the advantage of a long-term follow-up and a relatively large population; however, due to the de-identified nature of the dataset, we were unable to assess patient-reported outcome measures. To gain insights into factors that mediate the risk of lumbar spine reoperation, it is necessary to conduct a longitudinal prospective study incorporating standardized assessments of pain, low back-related disability, quality of life, and pain medication utilization [55, 56].

### Strengths and limitations

Strengths of this study included having a large propensity matched population, multidisciplinary author team, and registered protocol. Our study findings provide markers of validity in alignment with prior research. For instance, the incidence of reoperation in our usual care cohort was approximately 14%, a value slightly higher than prior estimates (up to 12% within 4 years post-discectomy [1–4]), which is justifiable given we only included patients with LSR. Furthermore, our cumulative incidence graph revealed an initial curvilinear increase in reoperation, suggesting that we succeeded in including patients with a heightened likelihood of reoperation [6, 24]. Finally, our study also highlights patients’ adherence to care in the SMT cohort, as evidenced by most patients having multiple SMT visits.

This study also has limitations. As in all observational studies, we are unable to infer causality from our findings. Between-cohort differences in unmeasured confounding variables may have influenced results, such as socioeconomic variables (e.g., unemployment [57]), patient-reported outcome measures of pain, disability or quality of life [31], length of preoperative symptoms before the primary discectomy [58], type of disc herniation (e.g., extrusion, sequestration; [38]), presence of adjacent segment degeneration [24], and lumbar spine range of motion [38], which were largely unavailable in the TriNetX dataset. It is also possible that variation in geographic region, surgeon specialty, or surgeon experience would influence the likelihood of reoperation [4]. Although we were unable to ascertain the exact type of discectomy per patient, one recent study found no difference in likelihood of reoperation among patients receiving open versus percutaneous lumbar discectomy [2]. As our dataset did not provide detailed patient-level data, we were unable to determine the length of time since the primary discectomy, although it was at least one year for all patients per our selection criteria. A 20-year data range was necessary to maximize sample size and allow for a lengthy follow-up window yet may have affected our findings considering changes in lumbar spine surgery practices over this period. The natural language processing tool we used has not been validated for the specific purposes of our study methods and it’s performance in this context is uncertain.

As a real-world study, patients in both cohorts used several types of pharmacologic and/or non-pharmacologic interventions prior to the index date of meeting our selection criteria, yet we were unable to examine their response to these forms of care. While it remains possible that the type of response to previous interventions influenced patients’ likelihood of reoperation, available evidence regarding this is limited [31, 57]. It is possible that cohorts had differential follow-up completeness, however, our required follow-up encounter, lengthy follow-up duration, and capacity for TriNetX to link patients’ encounters across included healthcare organizations minimized this potential variation.

Propensity score matching is an often-used method to adjust for confounding in observational studies, yet can paradoxically increase imbalance and bias especially when the model is misspecified [59]. In addition, propensity score matching prunes potentially relevant patients, thereby reducing available sample size [59]. While our propensity score density plot and post-matching SMD values for matched confounders suggested adequate matching [41, 60, 61], alternative strategies such as direct matching may have offered advantages [59].

This study focused on the endpoint of reoperation while other highly relevant clinical outcomes of changes in pain, disability, and quality of life could not be examined and may have differed between cohorts. Ideally, these measures would be controlled for and compared longitudinally from prior to the index date of inclusion, at the index date, and during care (SMT and usual care) for at least two years’ follow-up. Without these details, we cannot provide a comprehensive measure of effectiveness of SMT versus usual care in treatment of LSR following lumbar discectomy.

The results of this study may only generalize to adults attending US academic healthcare organizations as other regions may have different management strategies for LSR after lumbar discectomy.

## Conclusions

This study found that adults receiving SMT for LSR at least one year after lumbar discectomy were less likely to undergo lumbar spine reoperation compared to those receiving usual care without SMT, a difference which persisted over two years’ follow-up. These findings highlight the potential role of SMT in reducing the likelihood of additional surgery in this patient population. However, prospective studies are needed to validate our findings and concurrently examine changes in pain, disability, and safety among those receiving SMT after discectomy.

### Electronic supplementary material

Below is the link to the electronic supplementary material.

Supplementary Material 1

## Data Availability

Minimal, aggregate, de-identified datasets used to report our key outcomes and plot cumulative incidence and propensity score density are available in the Figshare repository (10.6084/m9.figshare.24769767).

## Abbreviations

CIs: Confidence Intervals
CTSC: Clinical and Translational Science Collaborative
DE: Germany
ICD-10: International Classification of Diseases, 10th Edition
LSR: Lumbosacral Radiculopathy
MA: Massachusetts
NIH: National Institutes of Health
RR: Risk Ratio
SMD: Standardized Mean Deviation
SMT: Spinal Manipulative Therapy
STROBE: Strengthening the Reporting of Observational Studies in Epidemiology
UHCMC: University Hospitals Cleveland Medical Center
USD: United States Dollar
US: United States
VA: Veterans Affairs
