# Supplementary material for: Association between spinal manipulative therapy and lumbar spine reoperation after discectomy: a retrospective cohort study
Source: BMC Musculoskelet Disord. 2024 Jan 10;25:46. doi: 10.1186/s12891-024-07166-x (PMC10777506; doi:10.1186/s12891-024-07166-x)
Supplement: Supplementary file 1 — Supplementary Material 1 [file 12891_2024_7166_MOESM1_ESM.pdf]

Supplemental File

Table 1: Lumbosacral radiculopathy definition

Diagnosis Codes (ICD-10)	Definition
G54.4	Lumbosacral root disorders, not elsewhere classified
M54.16	Radiculopathy, lumbar region
M54.17	Radiculopathy, lumbosacral region
M54.18	Radiculopathy, sacral and sacrococcygeal region
M54.3	Sciatica
M54.4	Lumbago with sciatica
Abbreviations: International Classification of Diseases, 10th Edition (ICD-10)	

Table 2: Lumbar discectomy

ICD-10 Codes	Definition
0SB2 (ICD-10-PCS)	Lower joints / excision / lumbar vertebral disc
0SB4 (ICD-10-PCS)	Lower joints / excision / lumbosacral disc
62287 (CPT)	Decompression procedure, percutaneous, of nucleus pulposus, of IVD, any method using needle-based technique to remove disc material under fluoroscopic imaging or other form of indirect visualization, with discography and/or epidural injection(s) at the treated level(s), when performed, single or multiple levels, lumbar
63030 (CPT)	Laminotomy (hemilaminectomy), with decompression of nerve root(s), including partial facetectomy, foraminotomy and/or excision of herniated IVD, lumbar
239542002 (SNOMED)	Excision of lumbar IVD
Abbreviations: Current Procedural Terminology (CPT); International Classification of Diseases, 10th Edition Procedural Classification System (ICD-10-PCS); intervertebral disc (IVD); Systemized Nomenclature of Medicine (SNOMED)	

Table 3: Exclusion criteria

Code	Definition
Exclusions for both cohorts	
0SG0 (ICD-10-PCS)	Lower joints / fusion / lumbar vertebral joint
0SG1 (ICD-10-PCS)	Lower joints / fusion / lumbar vertebral joints, 2 or more
0SG3 (ICD-10-PCS)	Lower joints / fusion / lumbosacral joint
1004053 (CPT)	Osteotomy procedures on the spine (vertebral column)
1004064 (CPT)	Fracture and/or dislocation procedures on the spine (vertebral column)
1004085 (CPT)	Arthrodesis procedures on the spine (vertebral column)
1004123 (CPT)	Spinal instrumentation procedures on the spine (vertebral column)
M43.25 (ICD-10)	Fusion of spine, thoracolumbar region
M43.26 (ICD-10)	Fusion of spine, lumbar region
M43.27 (ICD-10)	Fusion of spine, lumbosacral region
M96.0 (ICD-10)	Pseudarthrosis after fusion or arthrodesis
Z98.1 (ICD-10)	Arthrodesis status
50172003 (SNOMED)	Lumbar spinal fusion
Exclusions for the usual medical care cohort only	
98940, 98941, 98942 (CPT)	Chiropractic spinal manipulative therapy
Abbreviations: Current Procedural Terminology (CPT); International Classification of Diseases, 10th Edition (ICD-10); ICD-10 Procedure Coding System (ICD-10-PCS); Systematized Nomenclature Of Medicine (SNOMED)	

Table 4: Variables controlled for in propensity score matching

Variable/Code	Description
Age	Age at index date (enrollment)
Tests	
39156-5	Body mass index (LOINC)
Diagnoses (ICD-10)	
F17	Nicotine dependence
M43.16	Spondylolisthesis, lumbar region
M43.17	Spondylolisthesis, lumbosacral region
Abbreviations: International Classification of Diseases, 10th Edition (ICD-10), Logical Observation Identifiers Names and Codes (LOINC)	

Table 5: Lumbar spine surgeries

ICD-10 Codes	Definition
0SW0 (ICD-10-PCS)	Lower joints / revision / lumbar vertebral joint
0SW4 (ICD-10-PCS)	Lower joints / revision / lumbosacral disc
0SW3 (ICD-10-PCS)	Lower joints / revision / lumbosacral joint
0SB0 (ICD-10-PCS)	Lower joints / excision / lumbar vertebral joint
0SB2 (ICD-10-PCS)	Lower joints / excision / lumbar vertebral disc
0SB3 (ICD-10-PCS)	Lower joints / excision / lumbosacral joint
0SB4 (ICD-10-PCS)	Lower joints / excision / lumbosacral disc
0SG0 (ICD-10-PCS)	Lower joints / fusion / lumbar vertebral joint
0SG1 (ICD-10-PCS)	Lower joints / fusion / lumbar vertebral joints, 2 or more
0SG3 (ICD-10-PCS)	Lower joints / fusion / lumbosacral joint

00NY (ICD-10-PCS)	Central nervous system and cranial nerves / release / lumbar spinal cord
22533 (CPT)	Arthrodesis, lateral extracavitary technique, including minimal discectomy to prepare interspace (other than for decompression); lumbar
22558 (CPT)	Arthrodesis, anterior interbody technique, including minimal discectomy to prepare interspace (other than for decompression); lumbar
22586 (CPT)	Arthrodesis, pre-sacral interbody technique, including disc space preparation, discectomy, with posterior instrumentation, with image guidance, includes bone graft when performed, L5-S1 interspace
22612 (CPT)	Arthrodesis, posterior or posterolateral technique, single interspace; lumbar (with lateral transverse technique, when performed)
22630 (CPT)	Arthrodesis, posterior interbody technique, including laminectomy and/or discectomy to prepare interspace (other than for decompression), single interspace; lumbar
22633 (CPT)	Arthrodesis, combined posterior or posterolateral technique with posterior interbody technique including laminectomy and/or discectomy sufficient to prepare interspace (other than for decompression), single interspace; lumbar
22857 (CPT)	Total disc arthroplasty (artificial disc), anterior approach, including discectomy to prepare interspace (other than for decompression), single interspace, lumbar
62287 (CPT)	Decompression procedure, percutaneous, of nucleus pulposus, of IVD, any method using needle-based technique to remove disc material under fluoroscopic imaging or other form of indirect visualization, with discography and/or epidural injection(s) at the treated level(s), when performed, single or multiple levels, lumbar
63005 (CPT)	Laminectomy with exploration and/or decompression of spinal cord and/or cauda equina, without facetectomy, foraminotomy or discectomy (eg, spinal stenosis), 1 or 2 vertebral segments; lumbar, except for spondylolisthesis
63017 (CPT)	Laminectomy with exploration and/or decompression of spinal cord and/or cauda equina, without facetectomy, foraminotomy or discectomy (eg, spinal stenosis), more than 2 vertebral segments; lumbar
63011 (CPT)	Laminectomy with exploration and/or decompression of spinal cord and/or cauda equina, without facetectomy, foraminotomy or discectomy (eg, spinal stenosis), 1 or 2 vertebral segments; sacral
63030 (CPT)	Laminotomy (hemilaminectomy), with decompression of nerve root(s), including partial facetectomy, foraminotomy and/or excision of herniated IVD, lumbar
63042 (CPT)	Laminotomy (hemilaminectomy), with decompression of nerve root(s), including partial facetectomy, foraminotomy and/or excision of herniated IVD, reexploration, single interspace; lumbar
63044 (CPT)	Laminotomy (hemilaminectomy), with decompression of nerve root(s), including partial facetectomy, foraminotomy and/or excision of herniated IVD, reexploration, single interspace; each additional lumbar interspace (List separately in addition to code for primary procedure)
63047 (CPT)	Laminectomy, facetectomy and foraminotomy (unilateral or bilateral with decompression of spinal cord, cauda equina and/or nerve root[s], [eg, spinal or lateral recess stenosis]), single vertebral segment; lumbar
63056 (CPT)	Transpedicular approach with decompression of spinal cord, equina and/or nerve root(s) (e.g., herniated IVD), single segment; lumbar (including transfacet, or lateral extraforaminal approach) (e.g., far lateral herniated IVD)
1036727 (CPT)	Laminectomy, facetectomy, or foraminotomy (unilateral or bilateral with decompression of spinal cord, cauda equina and/or nerve root[s] [eg, spinal or lateral recess stenosis]), during posterior interbody arthrodesis, lumbar
1029686 (CPT)	Insertion of interlaminar/interspinous process stabilization/distraction device, without fusion, including image guidance when performed, with open decompression, lumbar
1029687 (CPT)	Insertion of interlaminar/interspinous process stabilization/distraction device, without open decompression or fusion, including image guidance when performed, lumbar
C9757 (HCPCS)	Laminotomy (hemilaminectomy), with decompression of nerve root(s), including partial facetectomy, foraminotomy and excision of herniated IVD, and repair of annular defect with implantation of bone anchored annular closure device, including annular defect measurement, alignment and sizing assessment, and image guidance; 1 interspace, lumbar
178774008 (SNOMED)	Exploratory lumbar laminectomy

239542002 (SNOMED)	Excision of lumbar IVD
50172003 (SNOMED)	Lumbar spinal fusion
Abbreviations: Current Procedural Terminology (CPT); Healthcare Common Procedure Coding System (HCPCS); International Classification of Diseases, 10th Edition Procedural Classification System (ICD-10-PCS); intervertebral disc (IVD); Systemized Nomenclature of Medicine (SNOMED)	
